# Exploring the Genetic Landscape of Mild Behavioral Impairment as an Early Marker of Cognitive Decline: An Updated Review Focusing on Alzheimer’s Disease

**DOI:** 10.3390/ijms25052645

**Published:** 2024-02-24

**Authors:** Efthalia Angelopoulou, Christos Koros, Alexandros Hatzimanolis, Leonidas Stefanis, Nikolaos Scarmeas, Sokratis G. Papageorgiou

**Affiliations:** 1 1st Department of Neurology, Aiginition University Hospital, National and Kapodistrian University of Athens, 11528 Athens, Greece; angelthal@med.uoa.gr (E.A.); lstefanis@bioacademy.gr (L.S.); ns257@cumc.columbia.edu (N.S.); sokpapa@med.uoa.gr (S.G.P.); 2 1st Department of Psychiatry, Aiginition University Hospital, National and Kapodistrian University of Athens, 11528 Athens, Greece; alhatzi@med.uoa.gr; 3 Department of Neurology, Columbia University, New York, NY 10032, USA

**Keywords:** mild behavioral impairment, dementia, Alzheimer’s disease, polygenic risk scores, SNPs, genetic variation, APOE

## Abstract

The clinical features and pathophysiology of neuropsychiatric symptoms (NPSs) in dementia have been extensively studied. However, the genetic architecture and underlying neurobiological mechanisms of NPSs at preclinical stages of cognitive decline and Alzheimer’s disease (AD) remain largely unknown. Mild behavioral impairment (MBI) represents an at-risk state for incident cognitive impairment and is defined by the emergence of persistent NPSs among non-demented individuals in later life. These NPSs include affective dysregulation, decreased motivation, impulse dyscontrol, abnormal perception and thought content, and social inappropriateness. Accumulating evidence has recently begun to shed more light on the genetic background of MBI, focusing on its potential association with genetic factors related to AD. The Apolipoprotein E (APOE) genotype and the MS4A locus have been associated with affective dysregulation, ZCWPW1 with social inappropriateness and psychosis, BIN1 and EPHA1 with psychosis, and NME8 with apathy. The association between MBI and polygenic risk scores (PRSs) in terms of AD dementia has been also explored. Potential implicated mechanisms include neuroinflammation, synaptic dysfunction, epigenetic modifications, oxidative stress responses, proteosomal impairment, and abnormal immune responses. In this review, we summarize and critically discuss the available evidence on the genetic background of MBI with an emphasis on AD, aiming to gain insights into the potential underlying neurobiological mechanisms, which till now remain largely unexplored. In addition, we propose future areas of research in this emerging field, with the aim to better understand the molecular pathophysiology of MBI and its genetic links with cognitive decline.

## 1. Introduction

Dementia affects more than 50 million individuals globally, and it is estimated that its prevalence will almost triple by 2050, leading to a major public health issue [1]. Alzheimer’s disease (AD) is the most common cause of dementia, constituting 60–70% of all cases [2]. Neuropathologically, AD is characterized by the deposition of extracellular beta-amyloid plaques and intracellular tau neurofibrillary tangles [2].

The vast majority of AD cases are sporadic with an unclear multifactorial etiology, with both genetic and environmental factors contributing to AD development [2]. Genetic causes, including gene mutations in Presenilin 1 (*PSEN1*), Presenilin 2 (*PSEN2*), and amyloid precursor protein (*APP*), account for fewer than 5% of cases and are inherited in an autosomal dominant manner [2].

Given the lack of effective therapeutic strategies, current research is focusing on the earliest stages of AD, which may offer a window for potential intervention with disease-modifying treatment approaches. The identification of individuals at risk for AD dementia is a high priority of clinical trials and clinical practice. Despite the increasing accessibility to preclinical AD biomarkers, such as amyloid and tau positron emission tomography (PET) as well as amyloid beta, tau and phosphorylated-tau levels in the cerebrospinal fluid (CSF), screening among individuals with normal cognition remains expensive and ineffective [3]. Hence, there is a growing interest in identifying inexpensive, non-invasive, and easy-to-administer markers for the early phases of dementia and AD-related neurodegeneration [3,4].

Neuropsychiatric symptoms (NPSs), including depression, anxiety, apathy, agitation, irritability, disinhibition, hallucinations, and delusions, are very common manifestations of AD dementia, since up to 97% of AD patients display at least one NPS during the course of the disease [5]. NPSs have been related to increased mortality, worse quality of life, functional disability, increased caregiver burden and overall healthcare costs as well as a higher likelihood of placement in nursing homes [5]. The relationship between NPSs and cognitive decline is not linear, implying that at least partially diverse pathogenic mechanisms might underlie the cognitive and neuropsychiatric axis of neurodegenerative diseases [6].

Although NPSs have become more common as the disease progresses, they may occur at prodromal and preclinical stages, even before the manifestation of cognitive impairment [5,7]. NPSs, including depression and anxiety, might also be the presenting symptoms of AD [5,6]. While the clinical profile of NPSs in manifested AD is well characterized, our understanding of NPSs at the preclinical and prodromal stages of AD at a clinical and neurobiological level has been insufficient.

Mild behavioral impairment (MBI) is a recently described neurobehavioral syndrome in later life, being associated with an increased risk of cognitive impairment and incident dementia [8,9,10]. According to the International Society to Advance Alzheimer’s Research and Treatment (ISTAART) and the Alzheimer’s Association (AA), MBI is characterized by the emergence of impactful and sustained NPSs in non-demented individuals older than 50 years of age, which are not attributed to a pre-existing medical or psychiatric conditions, such as major depressive disorder, general anxiety disorder, or schizophrenia. MBI domains include affective dysregulation, impulse miscontrol, decreased motivation, abnormal perception or thought content, and social inappropriateness. These symptoms should constitute a clear change from baseline behavior or personality traits, observed by the individual or an informant. For the exclusion of transient behavioral alterations and reactions to life stressors, NPSs should persist for at least for six months consistently or intermittently. The severity of NPSs can vary, but they should cause some degree of impairment in the workplace, social interactions, or interpersonal relationships. For meeting MBI criteria, the individual should be functionally independent with normal cognition, subjective cognitive decline (SCD), or mild cognitive impairment (MCI) [3]. MBI accounts for the neurobehavioral pre-dementia axis, complementing the classical neurocognitive pre-dementia axis of MCI.

In this context, an MBI assessment may provide a useful, relatively easy, and inexpensive tool for identifying cognitively asymptomatic individuals at risk for cognitive decline. In this way, the pool of potential participants for clinical trials investigating preventive strategies or therapeutic interventions in the early stages of AD can be also enriched.

The MBI Checklist (MBI-C) is a validated instrument specifically developed to detect MBI symptoms and can be filled by an individual or a proxy informant. It contains 34 questions, rated on a severity scale from 0 (not present) to 3 (severe). The cut-off points of 8.5 and 6.5 in MBI-C have shown good specificity and sensitivity in SCI and MCI, respectively [3]. The Neuropsychiatric Inventory Questionnaire (NPI-Q) is another scale used to define MBI in several studies. NPI-Q was initially designed for patients with dementia and not community-dwelling functionally independent individuals; however, there is evidence showing a good correlation between MBI-C and NPI-Q [11].

Given their relatively low cost and increasing availability, the genetic predictors of neurodegenerative diseases are also gaining increasing attention. The Apolipoprotein E (APOE) e4 allele is the strongest genetic risk factor for AD, and compared to non-carriers, carrying one and two APOE e4 alleles increases the risk for AD 2–3 and 10–12 times, respectively [12]. Genome-wide association studies (GWAS) have revealed numerous additional genetic variants that may affect AD risk, but each of them are to a lesser extent compared to *APOE* e4 [13]. Polygenic risk scores (PRSs) represent the sum of risk alleles weighted by effect size for a particular disease or condition carried by a person, aiming to quantify the overall genetic predisposition to a specific complex trait or disease based on multiple genetic variants across the genome [13]. PRSs are calculated by combining information from numerous genetic markers, which are often single nucleotide polymorphisms (SNPs) and each with a small effect size on the condition of interest, identified via GWAS [13]. Since genetic variants are neither sufficient nor necessary to cause AD, a growing body of research is focusing on the identification of potential interactions between genetic variants and other clinical, biological, environmental, or lifestyle factors that affect the risk of developing AD.

Endophenotypes may represent clues to the underlying genetic architecture of neurodegenerative diseases such as AD, thereby aiding in a more successful genetic analysis [6]. Genetic variants account for about 53% of the overall phenotypic variability of late-onset AD [14], suggesting that the genetic component may play a pivotal role in the clinical differences observed throughout the disease course. For instance, a familial risk has been observed for the endophenotype of AD and psychosis, suggesting that a potential genetic component may contribute to this heterogeneity [15]. In this regard, the identification of preclinical or prodromal AD endophenotypes and our deeper understanding of their underlying neurobiology may help toward more effective and personalized therapeutic interventions at earlier stages.

There is extensive literature evidence on the underlying mechanisms and genetic architecture of NPSs in patients with clinically manifested neurodegenerative diseases, such as AD dementia, Parkinson’s disease (PD), Lewy body dementia (LBD), and frontotemporal dementia (FTD) [16]. Among older individuals with normal cognition, most genetic studies on psychiatric symptoms have focused on diagnosed psychiatric diseases, such as late-onset schizophrenia and delusional disorder [17]. However, the clinical features and underlying pathophysiology of psychiatric diseases differ from that of NPSs in AD and other neurodegenerative diseases. Negative symptoms and disorganized behavior and speech, which are core features of schizophrenia, do not characterize AD-related psychosis well [18]. Compared to the auditory hallucinations and organized delusions observed in schizophrenia, patients with AD-related psychosis display visual hallucinations and less complex delusions, often involving misidentification, abandonment, or theft [18]. Vegetative symptoms, such as sleep disturbances and changes in appetite, which are core features of major depressive disorder, might be secondary to other comorbidities of the elderly population [19].

The pathophysiology and genetic underpinning of NPSs at prodromal and preclinical phases of neurodegenerative diseases remains largely unclear. It has been hypothesized that the accumulation of abnormal protein aggregates could generate the psychiatric symptomatology, while other authors have proposed that NPSs may directly exert detrimental effects on brain function, thereby resulting in the clinical features of neurodegenerative diseases [20,21]. NPSs could also represent a reactive response to cognitive decline [20,21]. Finally, it has been suggested that NPSs and cognitive decline might etiologically share common genetic and/or environmental factors, or synergistic effects of biological factors with NPSs might elevate the risk of neurodegeneration [20,21].

In this context, research has only recently begun to shed more light on the genetic landscape of MBI as well as how MBI may interact with PRSs or specific genetic variants to affect the risk of cognitive decline. Elucidating the relationship between MBI and genetic variants related to AD may aid in our deeper understanding of the underlying pathophysiological mechanisms of this newly defined entity and how this is related to neurodegeneration and cognitive impairment. In addition, exploring the interplay between MBI and specific genetic variants or PRSs for AD may contribute to a more tailored risk assessment, earlier detection, and possibly more personalized intervention. Furthermore, MBI represents a promising screening tool to enrich study samples with participants displaying a higher risk of dementia, facilitating the design of clinical trials in the field of dementia prevention and therapy including those using PRSs or specific genetic variants.

Although the role of MBI as an early marker of AD has been already reviewed elsewhere [10], the underlying mechanisms of MBI remain unclear, and there is no recent review focusing specifically on the genetic factors associated with MBI. In this updated narrative review, we summarize and critically discuss available literature evidence on the genetic background of MBI with an emphasis on AD, aiming to explore the potential underlying neurobiological mechanisms. In addition, we propose future areas of research in this emerging field in an attempt to better understand the molecular pathophysiology of MBI and its genetic links with cognitive impairment.

For this purpose, we followed a systematic approach, searching Scopus and MEDLINE databases for clinical studies investigating the genetic factors associated with MBI with an emphasis on AD and no time restrictions. The search was performed between September 2023 and December 2023. We used the terms “mild behavioral impairment”, “MBI”, “behavioral symptoms”, “neuropsychiatric symptoms”, “affective”, “anxiety”, “depressive”, “depression”, “mood disorders”, “emotional”, “apathy”, “motivational”, “motivation”, “impulse control”, “psychotic”, “psychosis”, “delusions”, “delusional”, “hallucinations”, “MCI”, “mild cognitive impairment”, “cognitive decline”, “dementia”, and “Alzheimer’s disease” in various combinations. We screened search results in terms of the title and abstract and read the full form of relevant articles. Through the snowballing process, the bibliography of each relevant article was also screened for additional studies. We primarily included studies investigating the genetic factors associated with MBI focusing on dementia and AD. For discussion purposes, we were also focused on clinical studies investigating individual NPSs in AD and MBI in other neurodegenerative diseases such as PD or FTD as well as preclinical studies for exploring the potential implicated molecular mechanisms.

## 2. The Relationship between Apolipoprotein E (APOE) Genotype and Mild Behavioral Impairment (MBI)

Some of the first evidence comes from a study by Andrews and colleagues in 2018 among 1226 non-demented individuals, which aimed to investigate whether specific genetic variants related to AD dementia were associated with the altered likelihood of particular MBI domains [22]. In this study, among all the studied genetic factors, the *APOE* e4 allele was the only one related to a higher likelihood of affective dysregulation after adjustment for multiple comparisons, while no significant relationships were detected for the other MBI domains [22]. Hence, the *APOE* e4 allele might increase the risk for later-life affective dysregulation symptoms in the context of MBI during the preclinical stages of AD, although longitudinal evidence is needed to confirm this hypothesis. In addition, further evidence is needed regarding the relationship between the *APOE* genotype and specific subdomains of affective dysregulation, including depression, anxiety, and elation.

Although some studies have shown no associations between the *APOE* genotype and affective symptoms in AD, there is also evidence supporting the relationship between the *APOE* e4 allele and depression and anxiety in AD [6]. A meta-analysis among non-AD populations demonstrated that the presence of the *APOE* e4 allele was related to a higher risk of late-life depression [23]. In agreement with these results, *APOE* e4 was related to more severe late-life depressive symptoms and incident minor depression in a longitudinal study, even after the exclusion of participants who developed dementia over a nine-year follow-up period [24]. Low plasma Aβ42/Aβ40 has been associated with a higher risk of incident depression among only *APOE* e4 carriers in another study [25]. Increased atrophy of the temporal lobe has been linked to the presence of the *APOE* e4 allele [26] and a higher risk of incident major depression irrespective of cognitive decline [27]. These findings suggest that medial temporal atrophy and amyloid beta pathology might mediate the relationship between APOE e4 and late-life depression.

Concerning the potential neurobiological mechanisms in mice, the *APOE* e4 allele has been associated with acute stress-induced depression-like behaviors during aging, accompanied by impaired glucose metabolism in the hippocampus and prefrontal and temporal cortex, decreased levels of adenosine triphosphate (ATP), and mitochondrial dysfunction in astrocytes [28]. Depressive symptoms in *APOE* e4 carriers have also been related to lower levels of hsa-microRNA (miR)-107 [29], whose expression is reduced in the early stages of AD [30]. The β-site APP-cleaving enzyme 1 (BACE1), which is implicated in amyloid beta plaque formation, is one of the primary target proteins of hsa-miR-107 [31]. Hence, has-miR-107 might represent one of the potential underlying mechanisms linking *APOE* e4 with depression and AD. Collectively, it could be speculated that later-life depressive symptoms in the context of MBI may be associated with the *APOE* e4 allele, although further evidence is needed to replicate these findings.

Regarding the relationship between APOE e4 and anxiety, a recent study demonstrated that among patients with MCI, anxiety was less common in *APOE* e4 carriers compared to non-carriers [32]. In addition, an interaction between the *APOE* e4 allele with both anxiety and depression was observed for conversion to dementia only in unadjusted analyses [32]. In male mice, the *APOE* e4 allele has been related to enhanced anxiety-like behavior in aged animals but not in younger ones [33]. Interestingly, the *APOE* e4 allele was related to the impaired suppression of plasma cortisol concentration after dexamethasone administration [33]. This finding could be linked to the disrupted cortisol-mediated modulation of glucose metabolism in the hippocampus observed in patients with AD [34] and the altered sensitivity of peripheral blood mononuclear cells to dexamethasone [35]. In this context, among non-demented individuals, *APOE* e4 carriers display more pronounced atrophy in the amygdala compared to *APOE* e3/e3 individuals [36], although there is also evidence not confirming these results [33]. Interestingly, the *APOE* e4 allele may also interact with RNF219/G variants to affect anxiety levels in women with MCI [37]. Ring finger (RNF) proteins have been implicated in myelin production, synaptic stability, and ubiquitin system regulation [37]. Hence, there is some evidence that *APOE* e4 could be possibly associated with higher MBI anxiety levels among older individuals. The potential synergy between the *APOE* and *RNF219* genotype on affective dysregulation should be also investigated.

No significant relationship has been observed between the *APOE* genotype and the other MBI domains, including decreased motivation, psychosis, impulse dyscontrol, and social inappropriateness [22]. There are conflicting results about the association between the *APOE* e4 allele and apathy in AD [6]. Regarding impulse dyscontrol, some cross-sectional studies have shown that the *APOE* e4 allele is related to agitated behavior in patients with dementia [38], although longitudinal evidence has not confirmed these findings [6]. A meta-analysis among individuals with MCI and AD dementia found no significant association between *APOE* e4 carriership or homozygosity with the presence of apathy, agitation, anxiety, depression, and irritability [39]. Finally, the role of the *APOE* genotype on social cognition and inappropriateness, especially in preclinical AD populations, remains largely unknown.

The effect of the *APOE* genotype on the association between MBI and cognition was investigated by Creese and colleagues in 2021 among 2750 individuals [40]. According to this study, the impact of MBI stratification on the link between PRSs for AD and cognition became weaker after controlling for the *APOE* genotype [40]. These results suggest that the *APOE* e4 allele might at least partially mediate the relationship between MBI and cognitive function. Another study based on the same dataset demonstrated that the relationship between psychosis and incident cognitive impairment, as assessed by the MBI-C, was modified by the *APOE* genotype [41]. In this study, cognitive decline was determined via the progression of the Informant Questionnaire on Cognitive Decline in the Elderly (IQCODE) from <3.6 at baseline to >3.6 at annual re-evaluations with a maximum follow-up of 5 years [41]. More specifically, among carriers of at least one *APOE* e4 allele, psychosis was related to a greater hazard for cognitive decline, and a significant interaction was also observed between psychosis and *APOE* allele status [41]. This interaction persisted after excluding cases with hallucinations and only keeping those with delusions [41]. On the contrary, no interaction was observed in terms of the hazard for cognitive decline between psychosis and PRSs for AD, which included the *APOE* genotype together with > 83,500 SNPs [41]. A post-hoc analysis using PRSs that included only 22 SNPs revealed similar results as shown in the main analysis of the APOE mentioned above [41]. Collectively, these results suggest that the *APOE* e4 allele may be the primary genetic driver of the relationship between MBI psychosis and cognitive decline. Hence, it can be hypothesized that during the prodromal phase of AD, psychotic features might be more strongly related to incident cognitive decline among those carrying at least one *APOE* e4 allele.

Although psychotic manifestations are not common in prodromal phases of cognitive impairment, their presence may be associated with the greatest risk for dementia, compared to the other MBI domains [42]. Existing evidence on the association between the *APOE* e4 allele and psychosis in AD is inconsistent [6]. The *APOE* e4 allele and Lewy body pathology are more common in AD patients with hallucinations [43], suggesting that the *APOE* e4 allele might mediate cognitive impairment in the AD endophenotype with hallucinations, possibly via Lewy body neuropathology [43]. In addition, a longitudinal study has shown that the *APOE* e4 allele is linked to more severe worsening of delusions and hallucinations in later life among patients with schizophrenia, while this relationship was not detected in younger ages [44]. The *APOE* e4 allele was also associated with a higher risk of schizophrenia diagnosis in older adulthood [44]. As cognitive function was not investigated in this study, longitudinal evidence incorporating neuropsychological assessment would be particularly useful to clarify if this subgroup exhibited a higher likelihood of preclinical AD.

Another recent study, based on the longitudinal evidence from the National Alzheimer’s Coordinating Center (NACC) among 3932 non-demented individuals, aimed to investigate the potential relationship between decreased motivation and incident dementia and the possible modification by the *APOE* genotype [45]. Decreased motivation was defined by NPI-Q subscores of apathy > 0 in two annual consecutive evaluations [45], based on the published algorithm by Sheikh and colleagues in 2018 [46]. In this study, the apathy group displayed two times more rapid progression to dementia compared to the groups with no NPSs and no apathy [45]. Although apathy was related to a higher progression rate to dementia among all *APOE* genotypes, the contribution of apathy to dementia risk was the highest among *APOE* e3 carriers [45]. In this context, an additive interaction between apathy and the *APOE* e4 allele has been previously demonstrated for dementia risk among individuals with MCI [32,47]. Therefore, dementia risk attributed to apathy seems to be lower among *APOE* e4 carriers, potentially due to the greatest contribution of *APOE* e4-related pathways to the progression of neurodegeneration. Further evidence is needed in order to clarify if there is a synergistic interaction between apathy and the *APOE* e4 allele in affecting the risk of cognitive decline.

Based on the same database, another recent study indicated that affective dysregulation among non-demented individuals was associated with an increased risk of incident dementia, compared to no NPSs, and this relationship was stronger among *APOE* e4 non-carriers [48]. Affective symptoms in MCI have been linked to a higher risk of progression to dementia [49]. Furthermore, compared to e4 carriers, depressive symptoms have been related to an increased progression rate to MCI among older individuals with normal cognition and carrying no e4 allele [50]. These findings suggest that affective dysregulation might have a less significant contributing role in increasing the risk of cognitive decline among *APOE* e4 carriers due to the relatively large effect of the APOE e4 allele in dementia risk.

**Figure 1:**
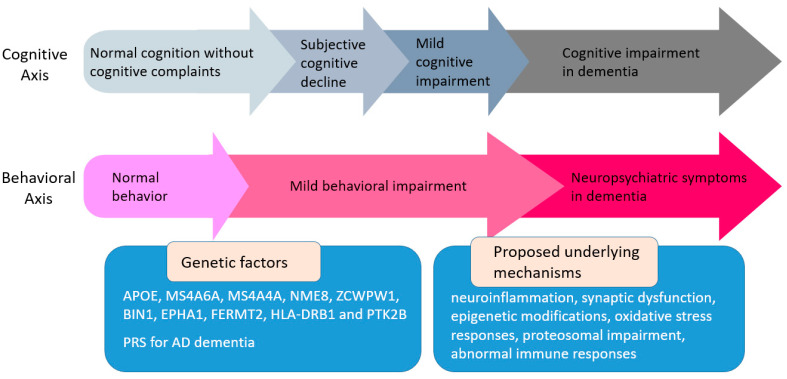
Alzheimer’s disease (AD)-related genetic factors possibly associated with mild behavioral impairment (MBI).

In summary, existing literature evidence suggests that the *APOE* e4 allele may be associated with affective dysregulation, while there is insufficient evidence to support its relationship with other MBI domains. Psychotic features may be more strongly related to dementia risk among *APOE* e4 carriers compared to non-carriers. On the contrary, the contribution of the MBI domains of apathy and affective dysregulation on dementia risk seems to be stronger among *APOE* e4 non-carriers. Taken together, although *APOE* e4 may be related to a higher risk of late-life affective dysregulation, there is likely no significant interaction between *APOE* e4 and this specific MBI domain in terms of dementia risk. On the other hand, there might be a possible synergistic effect of the MBI psychosis domain and *APOE* e4 allele on the risk of dementia, although future studies are needed to replicate these results (Figure 1, Table 1).

## 3. The Relationship between Other Alzheimer’s Disease (AD)-Related Genetic Factors and MBI

In addition to the APOE genotype, the relationship between individual genetic variants and MBI was investigated in a study by Andrews and colleagues in 2018. In particular, the association between individual MBI domains and 23 genetic loci known to be linked with AD risk was explored: *PICALM*, *HLA-DRB5*, *MEF2C*, *ABCA7*, *CELF1*, *CD33*, *DSG2*, *CD2AP*, *CR1*, *CLU*, *CASS4*, *BIN1*, *FERMT2*, *SORL1*, *NME8*, *INPP5D*, *EPHA1*, *MS4A4A*, *PTK2B*, *SLC24A4*-*RIN3*, *MS4A6A*, *MS4A4E*, and *ZCWPW1* [22]. Statistically significant relationships between genetic variants and MBI domains, other than those related to *APOE*, were detected only without adjustments for multiple comparisons [22]. In the following sections, we discuss the available literature evidence on the associations between genetic factors and MBI, aiming also to shed more light on the potential underlying neurobiological mechanisms.

### 3.1. MS4A Genetic Variants and Mild Behavioral Impairment (MBI)

Two SNPs in the *MS4A* genetic locus, named MS4A6A*G and MS4A4A*C, were associated with a lower risk of affective dysregulation [22]. *MS4A* gene products are considered to be implicated in neuroinflammatory responses by enhancing microglia activation and the production of pro-inflammatory cytokines [52,53]. Abnormal neuroinflammation has been linked to the longitudinal worsening of cognitive function among patients with AD [54]. NPS severity has been correlated with increased microglial activation across the AD continuum [55]. Excessive neuroinflammatory responses have also been related to anxiety and depressive, manic, and psychotic symptoms in several psychiatric disorders [56]. Importantly, the *MS4A* locus has also been associated with major depressive disorder [57]. Potential mechanisms include the dysregulation of the hypothalamic–pituitary–adrenal (HPA) axis, the immune cell glucocorticoid resistance leading to hyperactivation of the peripheral immune system, the disruption of the blood-brain barrier, and the activation of glial cells [58]. Increased levels of pro-inflammatory cytokines in the peripheral circulation, including interleukin (IL)-1β and the tumor necrosis factor alpha (TNF)-α, have been associated with a higher probability of major depressive disorder [59]. Therefore, the relationship between MBI and *MS4A* genetic locus suggests that aberrant neuroinflammatory responses might possibly play an important role in the pathophysiology of MBI, and especially affective dysregulation.

### 3.2. NME8 Genetic Variants and MBI

In the abovementioned study, the NME8*G allele was also related with a lower likelihood of decreased motivation [22]. In a separate analysis excluding patients with objectively normal cognition, NME8*G was inversely associated with social inappropriateness and affective dysregulation [22]. *NME8*, a member of the *NM23* family, has been identified as a cause of primary ciliary dyskinesia (PCD) [60]. Nucleoside diphosphate kinase (NDPK), which belongs to the NM23 family, is implicated in neuronal cell differentiation, proliferation, and neurite outgrowth, while its activity is reduced in the brain of patients with AD [61]. In AD, *NME8* locus polymorphisms have been related to the degree of cognitive impairment and occipital lobe and hippocampal atrophy as well as tau levels in the CSF [62]. However, the role of *NM23* gene products in neurodegenerative diseases and NPSs related to prodromal and preclinical phases remains unknown.

### 3.3. ZCWPW1 Genetic Variants and MBI

The genetic variant ZCWPW1*C has been inversely associated with psychotic manifestations and social inappropriateness in the abovementioned study [22]. However, in a supplementary analysis including only cognitively normal individuals, the association between ZCWPW1*C and psychotic features was lost [22]. *ZCWPW1* is involved in epigenetic regulation by recognizing histone modifications. Although the exact role of this genetic locus in neurodegeneration is unclear, it has been hypothesized to affect neurite elongation and protein degradation via proteosomal function and oxidative stress responses [63]. Nevertheless, its implications in terms of NPSs related to AD or other neurodegenerative diseases are unexplored. 

### 3.4. BIN1 Genetic Variants and MBI

In the abovementioned study, the genetic variant BIN1*G was associated with psychotic features [22]. Bridging integrator 1 (*BIN1*) constitutes the second most common genetic risk factor for AD [64]. The gene product of *BIN1* is implicated in endocytosis and the release of neurotransmitters [64]. In vitro evidence has shown that BIN1 may interact with tau, inhibit the extracellular uptake of tau seeds via endocytosis, and modulate its exosomal release [64]. BIN1 can mitigate tau pathology in specific brain regions of transgenic mouse models of AD, including the entorhinal/piriform cortex, hippocampus, and amygdala, thereby inhibiting neuronal cell death, synaptic loss, and neuroinflammation [64]. The underlying molecular mechanisms involved alterations in the expression of genes related to neuroinflammation and the promotion of microglia activation [64]. BIN1 can interact with amyloid beta to accelerate tau accumulation in patients with AD [65]. Furthermore, psychosis has been related to enhanced intra-neuronal tau accumulation in AD [66]. BIN1 has also been associated with major depressive disorder [57], whereas its implications in schizophrenia or other psychiatric conditions remain unexplored. Therefore, it can be speculated that BIN1 may mediate the link between tau neuropathology and psychosis in AD, although further evidence is needed especially for the presymptomatic stages.

### 3.5. EPHA1 Genetic Variants and MBI

The genetic variant EPHA1*C has also been related to MBI psychosis [22]. Ephrin type-A receptor 1 (EPHA1) is a receptor tyrosine kinase and is activated by ephrin-A, its membrane-bound ligand. Eph/ephrin-A signaling is implicated in several cellular functions, including axonal guidance, cell migration, synaptic plasticity, immune dysregulation, and neuroinflammation. *EPHA1* genetic variants have been linked to depressive symptoms in patients with AD dementia [67]. In Drosophila models, *EPHA1* mutations have been associated with hyperarousal, sleep reduction and increased locomotor activity as well as enhanced clock neuron excitability without affecting memory [68]. *EPHA1* genetic variants and impaired Eph/ephrin-A signaling have been associated with mild non-cognitive behavioral deficits, which might reflect the earliest stages of AD [68]. In this study, *EPHA1* mutations did not result in a neurodegenerative phenotype and did not affect the longevity of the animals [68], implying that they might impair neuronal activity independent of aging or neurodegeneration. Hence, it can be hypothesized that *EPHA1* genetic variants may contribute to MBI, by affecting the Eph/ephrin-A pathway and subsequently, several cellular mechanisms.

### 3.6. FERMT2 Genetic Variants and MBI

In supplementary analyses of the abovementioned study excluding participants with cognitive impairments, SNP FERMT2*C was inversely related to impulse dyscontrol [22]. Fermitin family homolog 2 (FERMT2), also known as kindlin-2, can interact with APP and regulate its metabolism, as well as modulate axon guidance, long-term potentiation, and synaptic connectivity in an APP-dependent manner [69]. *FERMT2* expression is also altered in male patients with schizophrenia [70]. Although the potential role of *FERMT2* variants in MBI is still unknown, based on the findings above, it might possibly affect synaptic connectivity and transmission.

### 3.7. HLA-DRB1 Genetic Variants and MBI

The genetic variant HLA-DRB1*T has been linked to impulse dyscontrol and psychosis among individuals with normal cognition [22]. The human leukocyte antigen (HLA)-DRB1 is implicated in histocompatibility and a wide range of immune responses. *HLA-DRB1* genetic variants have been associated with anxiety [71], affective distress [72], and schizophrenia [73] in non-AD populations. Furthermore, specific SNPs in *HLA-DRB1* have been related to the altered volume of the left posterior cingulate gyrus in a large sample of patients with MCI and normal cognition, suggesting their potential role especially in the early stages of AD [74]. Abnormalities in the posterior cingulate appear in the earliest phases of AD [75]. The posterior cingulate cortex is the brain region most strongly associated with the rate of cognitive decline across the AD spectrum [76]. This region is considered as the central hub of the default mode network, being inter-connected with many other networks. Default mode network abnormalities are observed in the early stages of AD, even before amyloid accumulation [77]. In addition, the posterior cingulate gyrus is strongly related to anxiety, depression, and agitation in AD [78]. Enhanced neuroinflammation, amyloid beta deposition, and brain activity have been observed in this brain area during a working memory task in patients with AD [79]. Collectively, it could be hypothesized that *HLA-DRB1* variants might be related to NPSs in the earliest stages of AD, with a potential functional role particularly in the posterior cingulate gyrus.

### 3.8. PTK2B Genetic Variants and MBI

In a supplementary analysis of the abovementioned study only among participants with cognitive impairment, SNP PTK2B*C was inversely related to affective dysregulation [22]. Proline-rich tyrosine kinase 2 (Pyk2), the gene product of *PRK2B*, is a Ca^2+^-activated non-receptor tyrosine kinase that is implicated in synaptic plasticity and hippocampal function [80]. Pyk2 activity is involved in both amyloid beta-induced deficits and tau-related pathology in vivo [80]. In mice, Pyk2 affects the anhedonia-like and anxiety-like phenotypes as well as dendritic spine morphology in the amygdala [81]. Hence, PTK2B might be related to affective dysregulation in the preclinical stages of AD by influencing synaptic function and amyloid and tau pathology.

Collectively, variants of the *MS4A4A* and *MS4A6A* genes have been inversely associated with affective dysregulation, while *ZCWPW1* variants have been inversely related to social inappropriateness and psychotic features. *BIN1* and *EPHA1* have been linked to psychosis, whereas *NME8* has been inversely associated with apathy. These findings suggest that MBI and cognitive impairment in AD may share a common genetic etiology. Another possible interpretation is that intermediate signaling pathways indirectly related to these genetic variants might underlie both conditions, resulting in both cognitive impairment and NPSs. The molecular mechanisms underlying these relationships are largely unknown, although neuroinflammation, abnormal immune responses, neurite outgrowth, neuronal excitability, synaptic connectivity, beta-amyloid metabolism, proteosomal function, oxidative stress, and epigenetic modifications might be involved.

## 4. The Relationship between Polygenic Risk Scores (PRSs) for AD and MBI

### 4.1. The Association between PRSs for AD and MBI

Until recently, it was unknown whether MBI was related to PRSs for AD. The study by Andrews and colleagues indicated that a higher PRS for AD, including the APOE e4 allele and 23 genetic loci, was associated with affective dysregulation [22]. However, no such association was detected following the exclusion of the *APOE* genotype, suggesting that this relationship was primarily driven by *APOE* e4 [22]. Another study by Creese and colleagues based on the data from 2529 individuals from the PROTECT study demonstrated that PRSs for AD were associated with impulse dyscontrol and apathy [82]. Notably, this relationship was observed only in the proxy informant MBI-C responses and not in the self-rated responses, highlighting the value of obtaining informants’ assessments even in the preclinical stages of dementia [82]. In this study, no association was detected between PRSs for AD and the depressive symptoms evaluated by the Patient Health Questionnaire 9 (PHQ-9) [82]. The different results between these two studies could be explained by the different methods used for capturing depressive symptoms, further emphasizing the importance of utilizing MBI-C as a multi-domain and specific instrument to detect the emergence of NPSs in later life.

A recent systematic review demonstrated that the T allele in the 3′-untranslated region (3′-UTR) polymorphism of the prion-like protein Doppel (*PRND*) gene, and increased baseline levels of the TNF-α and IL-6 were associated with a higher risk for apathy in AD [83]. The *PRND* gene product is the Dopell protein, whose functional role in AD remains unclear. The T allele in the 3′-UTR of the *PRND* gene has been associated with a greater cumulative behavioral burden and increased risk of irritability, agitation, and apathy among individuals with AD [84]. Further evidence is needed to investigate whether PRSs for inflammatory markers are linked to decreased motivation as well as the underlying *PRND*-related pathogenic mechanisms.

### 4.2. The Interaction between PRSs for AD and MBI on Cognition

A study including 4458 cognitively normal individuals older than 50 years of age from the PROTECT study aimed to investigate if the sample stratification by MBI affects the relationship between the PRSs for AD and global cognition [40]. In this analysis, PRSs for AD dementia were calculated based on the International Genomics of Alzheimer’s Project (IGAP), a GWAS for AD, and MBI was determined by an MBI-C total score > 0 [85]. PRSs for AD were associated with a worse global cognitive composite score, while after stratification, this relationship persisted only in individuals with MBI [40]. After control for a self-reported lifetime psychiatric diagnosis and PHQ-9 score ≥ 10, which is indicative of a current major depressive episode, this relationship remained significant [40]. The impact of MBI stratification on the link between PRSs for AD and cognition became weaker after controlling for the APOE genotype, indicating that APOE itself contributes to this MBI–PRS interaction in relation to cognition to some extent. In addition, this association was stronger among individuals above the age of 65 years with a higher MBI-C total score (≥6) in a post-hoc analysis [40]. The influence of PRSs on incident cognitive decline after stratification for different MBI domains remains unexplored. Collectively, these findings suggest that MBI may modify the relationship between PRSs for AD and cognitive ability.

Based on the above evidence, it can be hypothesized that among older individuals without cognitive impairment, MBI may characterize an endophenotype that more closely resembles prodromal AD. MBI was also proposed as a screening tool in clinical studies among older individuals at the prodromal or preclinical phases of AD, thereby contributing to a greater etiological homogeneity of the sample and providing higher power for detecting relationships between genetic variants with small effect sizes [40]. As heterogeneity is considered a significant challenge in relevant clinical studies for AD [86], MBI together with other clinical or biological markers including neuropsychological testing, neuroimaging, and fluid biomarkers might contribute to the better characterization and phenotyping of individuals at risk for cognitive decline. MBI assessment represents a low-cost, scalable, and simple strategy for enhancing enrollment in clinical trials for dementia. This approach will also allow for more personalized interventions at the prodromal or preclinical stages of AD.

It still remains largely unknown if MBI interacts with PRSs to increase the risk of dementia, since longitudinal studies with large sample sizes are needed to confirm this assumption. In this regard, another study whose data derived from PROTECT study demonstrated no interaction between MBI psychosis and PRSs for AD, regarding the risk for cognitive decline [41]. In this study, PRS tertiles were used, while in the previous study PRS underwent standardization for achieving a mean of 0 and a standard deviation of 1 for the statistical analysis [40]. In both cases, the PRSs included the *APOE* genotype for the main analyses [40,41]. Future studies are needed in order to clarify if the PRS may interact with MBI as a whole or the MBI domains to affect the risk of incident cognitive decline.

In summary, PRSs for AD may be associated with affective dysregulation, impulse dyscontrol, and apathy, while PRSs for AD have been associated with worse global cognition only among individuals with MBI. No significant interaction seems to exist between MBI psychosis and PRSs for AD in terms of the risk for cognitive decline, while evidence regarding the other MBI domains is missing. Longitudinal studies are needed in order to clarify the possible interactions between PRSs for AD and MBI regarding the risk for dementia.

## 5. Challenges and Limitations

The investigation of the genetic background of NPSs in terms of the broad syndrome of MBI, compared to that of individual NPSs, may inherently involve some limitations and challenges. Individual MBI domains, and clinical symptoms within the same MBI domain, may display distinct underlying neurobiological mechanisms, possibly corresponding to diverse genetic risks. Nevertheless, the investigation of MBI instead of individual NPSs offers a potentially significant advantage: MBI reflects a more comprehensive and global evaluation of a sum of even subtle behavioral changes of multiple domains, which might not be detectable if assessed by scales specifically designed for depression, apathy etc. or even clinical interviews aiming to identify signs of major depression or other psychiatric disorders. Ideally, genetic associations should be explored for both MBI as a global indicator of NPSs and separately for individual MBI domains.

The use of MBI or individual MBI domains as a dichotomous variable (presence/no presence) might also not be ideal for investigating the relationships between behavioral changes and genetic factors. For our deeper understanding of this link, it would be useful to additionally explore the severity of MBI or individual MBI domains in future studies.

Another important issue is the fact that the number of risk alleles included in PRS calculation may differ among various studies, thereby raising concerns about the generalizability and broad applicability of the findings. In this regard, diverse thresholds have been utilized for the calculation of PRSs for AD, ranging from SNPs revealed to be significant from GWAS to thousands of SNPs [87,88]. In the study by Creese and colleagues, >83,500 SNPs were utilized for the calculation of the PRSs used in the main analysis, while for the post-hoc analysis, only 22 SNPs were used [41]. As mentioned above, these different approaches resulted in different study results, which could be explained by the relatively large effect of the *APOE* genotype on the observed relationship. In the study by Andrews and colleagues, 23 genetic loci were used for the PRS calculation for AD in addition to the *APOE* genotype [22]. Varied findings among studies have also been previously demonstrated depending on the number of risk alleles used for PRS calculation [87,89,90]. Hence, the interpretation and comparison between the results of different studies require caution.

Regarding the *APOE* genotype, another issue is how the presence of the *APOE* e2 allele, and especially the *APOE* e2/e4 genotype, is methodologically approached. In this regard, it has been demonstrated that *APOE* e4 homozygosity is associated with the presence of MBI among individuals with SCD [51]. In the study by Andrews and colleagues, individuals with *APOE* e2/e4 genotype were excluded from the analyses for preventing conflation between the risk effect of *APOE* e4 and protective impact of *APOE* e2 [22]. Genetic studies in AD have often analyzed the *APOE* genotype as a dichotomized e4 allele status, not accounting for the number of e4 alleles usually due to the rarity of *APOE* e4 homozygosity.

Furthermore, the diverse tools used for defining MBI may critically affect the results of each study and contribute to heterogeneity. For instance, the two studies by Creese and colleagues used the MBI-C for defining MBI [40,41], whereas in the study by Andrews and colleagues, the NPI-Q was utilized [22]. MBI-C is an instrument specifically designed for MBI characterization among functionally independent non-demented older individuals. On the other hand, NPI-Q has been developed for patients with dementia, and some of the included items, such as wandering, are not ideal for the functionally independent population that MBI refers to. Importantly, the time frame of NPI-Q is significantly shorter than that of MBI-C (1 month versus 6 months respectively); hence, transient reactive conditions to stressful events are more likely to be included in the NPI-Q compared to MBI-C, possibly resulting in lower specificity. Therefore, the use of NPI-Q may result in the overestimation of MBI prevalence [3]. Although the use of two consecutive NPI-Q measurements increases the specificity for MBI characterization, there is no guarantee that NPSs exist during the period between these two time points [48]. Furthermore, for assessing symptoms of depression or apathy, NPI-Q contains few items, and it is not considered the ideal tool especially for these purposes [6].

Although MBI-C is a validated instrument for MBI, the optimal cut-off points, especially among cognitively normal individuals, have not been completely clarified and neither have the cut-off points for individual MBI domains [3]. Another crucial factor that may affect study results is how the MBI-C or other behavioral scales for MBI characterization are filled. Little overlap has been shown between self- and informant-rated responses in the MBI psychosis domain as assessed by MBI-C [41], while differences among PRSs and MBI have been detected between self-reported and informant-reported MBI-C responses [82]. Anosoagnosia is an important aspect that has not been investigated in terms of MBI [3], and it may affect MBI-C accuracy in the case self-reporting. Although the use of the MBI-C online version has been validated [91,92], the remote unsupervised completion of MBI-C might have resulted in potential misclassifications [40,41].

Access to a computer and internet were also some of the inclusion criteria in these studies by Creese and colleagues [40,41]. Hence, matters of digital literacy and ease with technology might have led to selection bias. Indeed, there was a relatively increased number of individuals with high education in these studies [40,41]. On the other hand, the use of online scales increases the sample sizes, since individuals with limited in-person access to study centers could also participate.

In addition, the methods used for the determination of prior psychiatric illness is another important issue. The self-reported medical history of a psychiatric disorder may have limited accuracy due to recall bias, and it has been suggested that a variety of tools should be rather used to capture NPSs [41]. For instance, in the PROTECT study, in order to exclude those with a previous history of a psychotic disorder, participants were additionally asked if they had prior psychotic experiences [41]. Another proposed approach is the additional use of medical records, although higher cost, consent issues, and practical challenges in data management are some of potential barriers [41]. The consideration of somatic comorbidities or medication use that might be related to NPSs among older adults should be also taken into consideration.

In the study by Andrews and colleagues, as previously described, the results were partially different in supplementary analyses excluding participants with MCI or those without MCI, but cognitive impairment was objectively evaluated via neuropsychological testing [22]. Separate genetic analyses among participants with normal cognition, SCD, and MCI would be of particular value.

The methods, scales, and cut-off points used to define cognitive decline over time could significantly influence the associations between MBI and genetic factors. In this regard, in a supplementary analysis, after the use of 3.3 in the IQCODE as a cut-off point for determining incident cognitive impairment instead of 3.6, the interaction between MBI psychosis and *APOE* allele status became insignificant [41]. Further work is needed for determining the optimal definition and assessment of cognitive decline over time. Furthermore, MCI constitutes a heterogeneous syndrome, including individuals with various forms of cognitive impairment (single- and multiple-domain and amnestic and non-amnestic MCI) as well as individuals with cognitive deficits that are “close” to the characterization of dementia, as determining functional impairment could be possibly partially subjective [48]. Therefore, future studies should also include analyses, whenever possible, among MCI subgroups. Even though the outcome in longitudinal studies is specifically MCI or AD dementia, the different criteria used might also affect study results.

Large sample sizes and replication of findings in diverse cohorts are usually needed for genetic studies of PRSs and specific SNPs. Some studies investigating the relationship between genetic variants and NPSs in patients with AD or MCI have relatively small sample sizes, and the lack of associations might be due to limited power. Furthermore, as some studies have been conducted on highly educated individuals [40,41,48], the results might not be generalizable in other populations. The studies by Creese and colleagues [40,41], as well as Andrews and colleagues [22], have been performed in European populations. Hence, the results of the abovementioned studies may not be generalizable to non-European populations. In this context, MBI-affective dysregulation has been recently related to an increased risk of dementia among Black individuals compared to White ones [48]. Even among the same race or country, the frequency of genetic variants may vary, such as in the case of *APOE* e4 whose prevalence seems to depend at least partially on geographical gradient [93], which could affect epidemiological results.

Importantly, a single individual may display different NPSs at different times [6], and diverse combinations of NPSs might also be observed throughout the course of preclinical AD. As some NPSs may often co-exist with others, such as agitation with psychotic features, it would be crucial to identify the primary contributors among MBI symptoms and/or combinations of them to clinical decline [41] and their interactions with genetic variants. Because of the subtle nature of MBI symptoms and the challenges of capturing dynamic behavioral changes over time, recall bias may be a prominent limitation of cross-sectional studies.

The inherent limitation of a cross-sectional study design to establish causality as well as the fact that behavioral symptoms tend to wax and wane over time [39] further highlight the importance of longitudinal studies. Hence, prospective studies investigating the clinical trajectory of MBI and different MBI domains could aid in clarifying the complex interaction between genetic factors and NPSs as a preclinical marker of neurodegenerative diseases.

## 6. Future Perspectives

As mentioned above, in rare cases, AD is caused by mutations in *APP*, *PSEN1*, or *PSEN2* genes. Existing evidence on the NPSs among carriers of these genetic mutations at presymptomatic stages is scant. In this context, a higher prevalence of apathy, depression, disinhibition, irritability, and agitation was observed in mildly symptomatic AD cases caused by *APP*, *PSEN1*, or *PSEN2* mutations compared to non-carriers [94]. However, no differences in NPSs were detected between carriers and non-carriers at presymptomatic stages in this study except for depression, which was rather unexpectedly more common among non-carriers [94]. Another longitudinal study indicated that NPSs, including disinhibition, depression, agitation, and irritability, were related to the decline of brain metabolism among cognitively normal individuals carrying *APP*, *PSEN1*, or *PSEN2* mutations [95]. These findings suggest that NPSs might represent an early clinical marker of cognitive impairment in individuals carrying these gene mutations. It would be interesting for future studies to explore the prevalence, clinical features, and pathophysiology of MBI in preclinical stages of genetic forms of AD. Furthermore, the relationship between non-pathogenic variants of *APP*, *PSEN1*, and *PSEN2* genes and NPSs among cognitively normal individuals should be further investigated. Given the significance of *APP*, *PSEN1*, and *PSEN2* in AD pathogenesis, this knowledge will deepen our understanding of the genetic underpinning of MBI.

It would be Interesting for future studies to explore the relationship between MBI and “atypical” non-amnestic clinical syndromes associated with AD, such as posterior cortical atrophy (PCA), logopenic variant primary progressive aphasia (lvPPA), and frontal variants of AD (fvAD) including cases presenting with dysexecutive AD (dexAD) or behavioral AD (bvAD) and corticobasal syndrome (CBS) [96]. It could be hypothesized that MBI could be possibly more frequently related to fvAD compared to other clinical syndromes. However, further evidence incorporating genetic analyses may aid in clarifying these possible associations.

Importantly, in schizophrenia, cognitive deficits such as executive dysfunction may precede the onset of the first psychotic episode [97]. One of the first reviews of family studies demonstrated that the strongest evidence of neuropsychological deficits in relatives of patients with schizophrenia was in sustained attention, perceptual-motor speed, and abstraction and concept formation, corresponding to prefrontal, temporal-limbic, and attentional networks [98]. Offspring and siblings of individuals with schizophrenia carrying a higher genetic risk for the illness display deficits in attention and memory [99]. Adolescents with a high genetic risk for schizophrenia also show impaired executive function and verbal ability [100]. In this regard, several genetic factors associated with schizophrenia risk are linked to executive function [101]. For instance, SNPs in the gene coding for catechol-O-methyltransferase (COMT) that are related to schizophrenia risk have been implicated in dorsolateral prefrontal and anterior cingulate physiological responses, prefrontal–hippocampal connectivity, and working memory performance [102,103,104]. Furthermore, a variant of the gene coding for regulator of G protein signaling 4 (RGS4) has been associated with both schizophrenia [105] and a lower volume of the dorsolateral prefrontal cortex [106]. The gene coding for metabotropic type II glutamate receptor mGluR3, *GRM3*, which is associated with schizophrenia, has been related to worse verbal fluency and lower neuronal integrity in the dorsolateral prefrontal cortex [107,108]. Given the fact that impaired executive function may also appear in the early stages of AD, executive dysfunction and its links to NPSs should be also considered in future genetic studies in MBI.

The relationship between depressive symptoms in AD and the *APOE* genotype has been observed only in women in some studies [6,109]. Among *APOE* e4 carriers with moderate to severe AD, disinhibition has been shown to be more common and more severe in women compared to men [110]. Sex also affected the association between cognitive decline and the *APOE* e2 allele in non-Hispanic White participants in a recent study, with men displaying slower decline compared to women [111]. *APOE* e4 homozygosity among participants with MCI was also related to a greater NPS burden in women compared to men [112]. In addition, sex-specific genetic associations have been detected between psychiatric disorders and cognitive function and behavior [113]. In particular, the PRSs for schizophrenia in males displayed greater correlation with cognitive abilities, while the PRSs for autism in females exhibited a greater correlation with fluid intelligence [113]. Hence, stratification by sex might be useful in future studies examining potential interactions between genetic factors and MBI symptoms in preclinical AD.

Moreover, the *APOE* genotype might be related with cardiovascular risk factors in later life [6]. The *APOE* e4 allele has been associated with atherosclerosis, hyperlipidemia, ischemic cardiovascular disease, and subcortical white matter hyperintensities [6]. It has been consistently shown that cardiovascular disease may be related to depression among patients with dementia [114]. The detrimental impact of even subtle cardiovascular risk factors has also been hypothesized to be more prominent among individuals who carry at least one e4 allele [115]. In this context, the potential interaction between MBI and PRSs in terms of predisposition to cardiovascular risk factors, such as diabetes, hyperlipidemia, or hypertension, that have been associated with white matter hyperintensities [116] should be also investigated.

A large population-based study demonstrated that PRSs for white matter microstructural alterations in the brain as evaluated by diffusion tensor imaging (DTI) were associated with AD, major depressive disorder, and lacunar stroke [117]. In the same study, genetic variants in the *VCAN* gene were also related to the white matter microstructural integrity [117]. Versican (VCAN) is a versatile protein, being implicated in signaling pathways regulating cell growth, motility, differentiation, and connectivity with the extracellular matrix [117]. Hence, white matter alterations may share a common genetic component with both depression and AD, and their role in MBI should be further explored.

In order to better understand the underlying molecular mechanisms of MBI and its relationship with neurodegeneration, future studies should focus on investigating the relationship between MBI and polygenic risk scores for specific disease-related pathways, such as pathways related to amyloid beta clearance, immune response, mitochondrial dysfunction, endocytosis, inflammatory activation, and protein-lipid and cholesterol metabolism [90,118]. AD is characterized by significant heterogeneity in terms of its clinical features as well as related atrophy and neuropathology [118]. For instance, possible AD subtypes have been suggested based on specific AD-related biomarkers, reflecting beta-amyloid or tau pathology [118]. MBI has been associated with amyloid and tau burden in PET in some but not all studies [3,119]. The identification of disease subtypes at a neurobiological level, especially at the preclinical stages based on the genetic background, might aid in the stratification of patients in clinical trials according to the underlying disease-related mechanism of the candidate drug.

In the same vein, several genetic variants related to AD are expressed in specific cell-types, including astrocytes and microglia, and based on this concept, cell-type specific polygenic risk scores have also been recently developed for AD. In this context, it has been recently demonstrated that among cognitively unimpaired older individuals, astrocytic PRSs for AD are linked to the beta-amyloid burden, while microglial PRSs for AD are linked to both beta-amyloid and tau accumulation. Given the association between neuroinflammation and NPSs across the AD spectrum, future studies should also investigate the relationship between glial PRSs for AD and MBI [55].

In PD, available evidence on the potential relationship between genetic factors and MBI is scant. A recent study among 146 patients with PD demonstrated that carriers of at least one Met allele of the p.Val66Met (G758A, rs62265) variant at position 66 in the exon 11 of the brain-derived neurotrophic factor (*BDNF)* gene displayed a higher likelihood of MBI, as assessed by MBI-C [120]. More specifically, compared to Val homozygotes, Met carriers exhibited two times increased probability of having MBI and elevated total MBI-C scores, after adjustment for Montreal Cognitive Assessment (MoCA) and Unified Parkinson’s Disease Rating Scale (UPDRS) part III scores [120]. Concerning the different MBI domains, Met carriers also had more severe psychotic and affective symptoms, compared to non-carriers [120]. A correlation between plasma BDNF levels and aggressiveness has been indicated in individuals with amnestic MCI and AD dementia [121]. Given the association between the *BDNF* gene variants and MBI in patients with PD, it could be hypothesized that BDNF gene variants might be related to MBI in preclinical AD populations too.

Future research should also focus on genetic factors associated with NPSs in manifested neurodegenerative diseases, including AD, PD, and FTD. For instance, the 102T/C polymorphism in the 5- hydroxytryptamine (HT) 2A receptor (*5-HT2AR*) gene has been associated with psychotic symptoms in patients with AD [122]. The rs2734849 polymorphism in the ankyrin repeat and kinase domain containing 1 (*ANKK1*) gene may be related to psychotic symptoms in patients with PD [123]. In FTD, it has been demonstrated that the *BDNF* Val66Met allele may be related to a lower burden of depressive symptoms [124]. In patients with behavioral variant of FTD (bvFTD), serotonin transporter length polymorphic region (*5-HTTLPR*) polymorphisms may be associated with altered volumes in brain regions implicated in socioemotional behavior, which is considered as representing a developmental or disease-related compensation for the altered serotonergic activity [125]. Such polymorphisms may be also related to MBI and should be further investigated.

Another crucial challenge is the complex etiology of neurodegenerative diseases, which is attributed not only to genetic but also environmental factors [126]. The investigation of the relationship between specific genetic variants and complex conditions such as MBI, might rather be an oversimplified approach. A recent study among individuals with amnestic MCI and AD dementia demonstrated that nutritional status as assessed by the Mini Nutritional Assessment (MNA) mediated the relationship between *APOE* e4 and apathy, hallucinations, and aberrant motor activity [127]. Future research should also focus on potential environmental and lifestyle factors related to MBI as well as possible gene-environment interactions.

The potential role of epigenetics underlying the pathophysiology of MBI and its connection to neurodegeneration should be also explored. DNA methylation biomarkers possibly affecting the risk for AD have been recently developed via the integration of blood and genome methylome data [128]. Sex-specific differences have been identified in the DNA methylation patterns affecting the risk of depression among patients with late-onset AD [129]. The relationship between DNA methylation biomarkers with affective dysregulation or other MBI domains could shed more light on the epigenetic mechanisms underlying MBI.

A systematic review has demonstrated that apathy progression in AD is related to specific premorbid personality characteristics, including higher neuroticism and lower levels of agreeableness [83]. It would be interesting for future studies to explore the potential associations between PRSs for personality traits, such as extraversion and neuroticism [130], and MBI or individual MBI domains.

Interestingly, it has been demonstrated that PRSs for major depressive disorder is related to the development of depression in patients with late-onset AD [131]. It would be useful for future studies to examine the association between PRSs for major depression disorder and MBI and especially the domain of affective dysregulation. In accordance, PRSs for schizophrenia [132] might affect the risk of the MBI domain of abnormal perception/thought content.

It is of note that existing studies have focused on the relationship between MBI and genetic factors related to AD among individuals with normal cognition, SCD, or MCI. However, the potential association between MBI and other genetic factors remains largely unexplored, including genes related to other forms of dementia such as FTD. FTD disproportionately appears in younger patients, and it represents the fourth most frequent dementia subtype, with 15–30% of cases caused by autosomal dominantly inherited gene mutations [133]. Since early core symptoms of FTD include behavioral changes, such as apathy or disinhibition, research criteria have been recently proposed for the prodromal bvFTD, termed “mild behavioral and/or cognitive impairment in bvFTD” (MBCI-FTD) [133]. This study was based on cohorts of individuals carrying pathogenic mutations known to cause FTD, such as *MAPT*, *C9ORF72*, and *PRGN*, and individuals with autopsy-confirmed FTD [133]. The seven core characteristics for MBCI-FTD were the following: apathy without moderate-severe dysphoria, irritability/agitation, disinhibition, decreased empathy/sympathy, gregariousness/joviality, simple or complex repetitive behavior as well as hyperorality/appetite alterations. Supportive characteristics included executive dysfunction or impaired naming with intact visuospatial skills and orientation, disrupted social cognition, and limited insight for behavioral or cognitive alterations. Possible MBCI-FTD was defined by the presence of three core characteristics or two core plus one supportive characteristic, while probable MBCI-FTD necessitated biomarker, neuroimaging, or genetic evidence [133]. It has been shown that MBI may progress to FTD too [134]. Although the expected proportion of older individuals developing FTD would be rather relatively low, the relationship between MBI and genetic factors associated with FTD should be also investigated for our better understanding of the genetic landscape of MBI. Large longitudinal studies would also aid in clarifying the interaction between genetic factors and MBI in the development of other dementia subtypes, vascular dementia, mixed dementia, Lewy body dementia, PD dementia, etc.

In addition, since MBI is a relatively recently described entity that is gaining increasing attention, the elucidation of its underlying biological mechanisms may also contribute to the improvement of the accuracy of the diagnostic criteria. For instance, individuals with MBI without amyloid accumulation as reflected by a higher risk of PRSs for amyloid pathology, positive PET scan, or lower amyloid beta levels in the CSF may display a much lower risk for cognitive decline compared to those with MBI but without amyloid pathology. Hence, the addition of indicators of amyloid pathology in the diagnostic criteria of MBI may significantly improve specificity regarding the risk for incident cognitive impairment. Future studies on MBI and the underlying neurobiology may prove very useful toward this direction.

## 7. Conclusions

Accumulating evidence has recently begun to shed more light on the potential genetic background of MBI, focusing on its possible association with the *APOE* genotype, other AD-associated genetic variants, and PRSs for AD. MBI assessment and genetic analysis represent relatively easy and low-cost screening methods, which might be used in combination to enrich the pool of participants and enhance the accuracy of determining preclinical AD. Although this research field is in its infancy, these initial findings aid in our better understanding of the neurobiological mechanisms underlying the newly defined entity of MBI, which may contribute to better stratification of preclinical AD and more personalized therapeutic interventions in future clinical trials.

## Figures and Tables

**Table 1:** Studies investigating the relationship between Alzheimer’s disease (AD)-related genetic factors and mild behavioral impairment (MBI).

Study	Country	Study Type	Aims	Study Participants	MBI Assessment	Main Results
Creese et al., 2021 [40] (data derived from a PROTECT study)	United Kingdom	Cross-sectional	To investigate whether the stratification of cognitively older individuals by mild behavioral impairment (MBI) affects the association between polygenic risk scores (PRSs) for Alzheimer’s disease (AD) and cognitive function	Non-demented individuals, ≥50 years of age, with access to a computer and internet, with available genotype, cognitive function and MBI-Checlist (MBI-C) data, without mild cognitive impairment (MCI), Parkinson’s disease (PD), or stroke (*n* = 4458)	MBI-C (cut-off points: zero and six in a post-hoc analysis)	PRSs for AD were associated with worse global cognition in the MBI group but not in the no MBI group The strongest association was observed in participants with more severe MBI (MBI-C score ≥ 6), aged ≥ 65 years After controlling for the apolipoprotein E (*APOE*) genotype, the impact of MBI stratification on the link between PRSs for AD and cognition became weaker After control for self-reported lifetime psychiatric diagnosis and a Patient Health Questionnaire 9 (PHQ-9) score ≥10, this relationship remained significant
Creese et al., 2021 [40] (data derived from a PROTECT study)	United Kingdom	Cross-sectional	To examine the relationship between PRSs for AD and MBI domains using both proxy informant and self-rated MBI-C responses	Non-demented individuals, ≥65 years of age, with access to a computer and internet, with available genotype, and MBI-C data (*n* = 2529)	MBI-C (measured by proxy informants and self-ratings)	PRSs for AD were associated with a higher risk of apathy and impulse dyscontrol only in proxy MBI-C responses but not in the self-reported MBI-C responses No association was detected between PRSs for AD and PHQ-9
Creese et al., 2023 [41] (data derived from a PROTECT study)	United Kingdom	Longitudinal	To investigate the relationship between MBI psychosis and incident cognitive impairment [annually assessed by Informant Questionnaire on Cognitive Decline in the Elderly (IQCODE)] and whether this relationship was modified by gender and genetic risk for AD (*APOE* e4)	Non-demented individuals, ≥50 years of age, with access to a computer and internet, with available genotype, and MBI-C data, without MCI, and IQCODE < 3.6 at baseline, without PD, epilepsy, multiple sclerosis, or stroke (*n* = 2750)	MBI-C (the only domain of psychosis used in this study, with the cut-off of >0 in at least one of the five items of the MBI-C that are related to psychosis)	The presence of MBI psychosis was associated with a greater hazard of cognitive decline compared to the absence of MBI Among *APOE* e4 carriers, MBI-psychosis was related to a higher hazard for cognitive decline compared to the absence of psychosis, while among non-*APOE* e4 carriers, the relationship was not significant There was an interaction between MBI psychosis and the *APOE* e4 allele affecting the hazard for cognitive decline, even after controlling for non-psychosis MBI After excluding the cases with hallucinations and keeping only those with delusions, the interaction persisted There was no interaction between MBI psychosis and PRSs for AD regarding the hazard for incident cognitive impairment A post-hoc analysis using PRSs including only 22 SNPs revealed similar results as shown in the main analysis of *APOE*
Nathan et al., 2020 [51] (data derived from the National Alzheimer’s Coordinating Center (NACC))	USA	Longitudinal	To cross-sectionally determine the frequency of *APOE* e4 homozygosity among individuals with subjective cognitive decline (SCD), stratified by MBI status	Non-demented older individuals with normal cognition but with SCD (*n* = 5005)	Neuropsychiatric Inventory Questionnaire (NPI-Q) according to the published algorithm by Sheikh and colleagues, 2018 [46]	Among older adults with SCD, *APOE* e4 homozygosity was more frequently seen in the MBI+ group.
Andrews et al., 2018 [22] (data derived from the Personality and Total Health Through Life project (PATH))	Australia	Cross-sectional	To investigate if PRSs for AD and specific genetic variants that are associated with a higher risk for AD have shared genetic factors related to MBI	Non-demented older individuals of European ancestry, ≥60 years of age, without *APOE* e2/e4 genotype and with normal cognition, or MCI (*n* = 1226)	NPI-Q (according to the published algorithm by Sheikh and colleagues, 2018 [46])	*APOE* e4 allele and higher PRSs (including *APOE* genotype) were associated with a higher likelihood of affective dysregulation The relationship between PRSs and affective dysregulation did not remain significant after the exclusion of the *APOE* genotype *MS4A4A*-rs4938933*C and *MS4A6A*-rs610932*G were associated with a lower likelihood of affective dysregulation (only without adjustments for multiple comparisons) *ZCWPW1*-rs1476679*C was associated with a lower likelihood of social inappropriateness and abnormal perception/thought content (only without adjustments for multiple comparisons) *BIN1*-rs744373*G and *EPHA1*-rs11767557*C were associated with a higher likelihood of abnormal perception/thought content (only without adjustments for multiple comparisons) *NME8*-rs2718058*G was associated with a lower likelihood of decreased motivation (only without adjustments for multiple comparisons)
Vellone et al., 2022 [45] (data derived from the National Alzheimer’s Coordinating Center (NACC))	USA	Longitudinal	To investigate whether MBI apathy is associated with progression to dementia, and if this relationship is modified by sex, race, cognitive diagnosis, and *APOE* genotype	Non-demented individuals with normal cognition or MCI, without past psychiatric, developmental, or neurological conditions, including post-traumatic stress disorder, bipolar disorder, schizophrenia, obsessive-compulsive disorder, anxiety, depression, Down syndrome, Huntington’s disease, or PD, and with available data for *APOE* genotype, cognitive status, age, race, and years of education (*n* = 3932)	NPI-Q according to the published algorithm by Sheikh and colleagues in 2018 [46] at two consecutive annual visits (only the domain of apathy was investigated in this study; the MBI apathy group included participants with NPI-Q subscore for apathy > 0 in both visits, and no prior psychiatric diagnosis; the NPS apathy group included participants with NPI-Q subscores for apathy > 0 in the first visit without considering the psychiatric history)	The progression to dementia was two times faster in the MBI apathy group compared to that of no NPSs and no apathy The contribution of MBI apathy to the risk of dementia was higher in individuals with normal cognition than those with MCI The contribution of MBI apathy to the risk of dementia was higher in the group of non-*APOE* e4 carriers
Ebrahim et al., 2023 [48] (data derived from the National Alzheimer’s Coordinating Center (NACC))	USA	Longitudinal	To investigate the longitudinal relationship between MBI affective dysregulation and incident dementia	Non-demented individuals with normal cognition or MCI, without past psychiatric or neurodevelopmental disorders, and with available data for *APOE* genotype, cognitive status, age, race, and years of education (*n* = 4984)	NPI-Q according to the published algorithm by Sheikh and colleagues in 2018 [46] at two consecutive annual visits (MBI affective dysregulation domain was defined as the NPI-Q subscore for depression, anxiety, or elation > 0 in both consecutive visits)	MBI affective dysregulation was associated with an increased risk of incident dementia compared to no-NPS Interaction analyses demonstrated that MBI affective dysregulation was related to an increased risk of incident dementia in *APOE* e4 non-carriers compared to carriers

## Data Availability

No new data were created or analyzed in this study. Data sharing is not applicable to this article.
